# Supplementary material for: Albumin Stabilized Fe@C Core–Shell Nanoparticles as Candidates for Magnetic Hyperthermia Therapy
Source: Nanomaterials (Basel). 2022 Aug 20;12(16):2869. doi: 10.3390/nano12162869 (PMC9414223; doi:10.3390/nano12162869)
Supplement: Supplementary file 1 [file nanomaterials-12-02869-s001.zip › nanomaterials-1802872-SI.pdf]

nanomaterials

Article

Albumin Stabilized Fe@C Core–Shell Nanoparticles as Candidates for Magnetic Hyperthermia Therapy

Maria Antonieta Ramírez-Morales [1,2,3], Anastasia E. Goldt [1,*], Polina M. Kalachikova[1,4], Javier A. Ramirez B. [1], Masashi Suzuki [5,6], Alexey N. Zhigach [7], Asma Ben Salah [5,6], Liliya I. Shurygina [8], Sergey D. Shandakov[8], Timofei Zatsepin [1], Dmitry V. Krasnikov [1], Toru Maekawa [5,6], Evgeny N. Nikolaev [1] and Albert G. Nasibulin [1,4,*]

Appendix A

Supplementary information

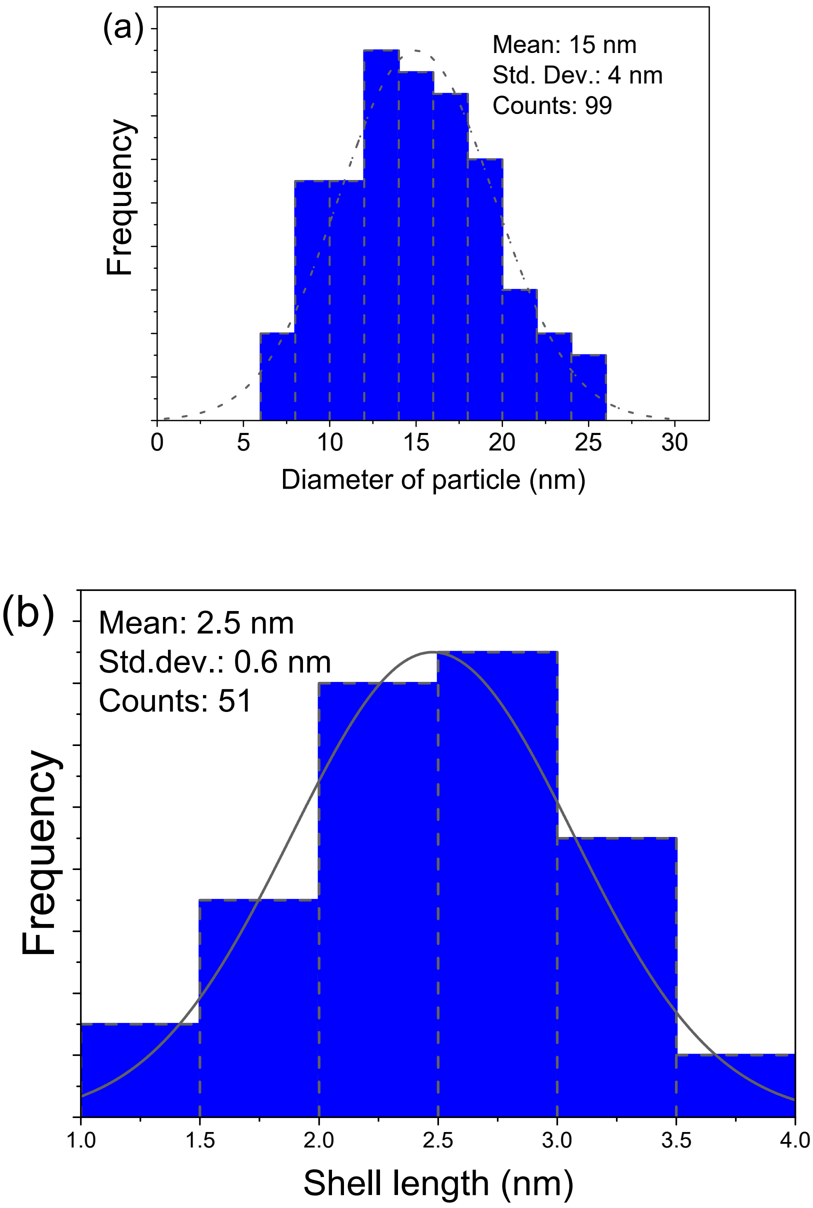

Figure S1 a) Particle size distribution and **b)** shell diameter of Fe@C nanoparticles

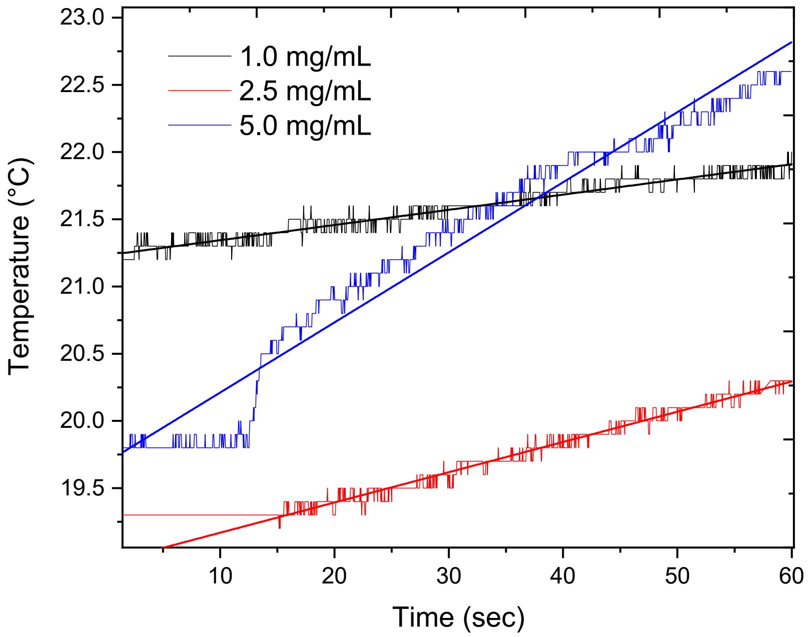

Figure S2. Temperature variation as a function of time for aqueous suspension of BSA-NPs with different concentrations of 1, 2.5 and 5.0 mg/ml at H = 752 Oe and frequency of *f* = 331 kHz.

Table S1. Parameters of linear equation fit of temperature behavior over time.

**Concentration, mg/mL**	**Linear equation**	**R^2**	**Standard error**
1.0	$y=1^{-5}x+21.23$	0.94	0.002
2.5	$y=2^{-5}x+18.95$	0.98	0.004
5.0	$y=5^{-5}x+19.69$	0.96	0.009
